# Metabolites Produced by the Endophytic Fungus *Aspergillus fumigatus* from the Stem of *Erythrophloeum fordii Oliv.*

**DOI:** 10.3390/molecules200610793

**Published:** 2015-06-11

**Authors:** Yu-Sheng Shi, Yan Zhang, Xiao-Zhong Chen, Ning Zhang, Yun-Bao Liu

**Affiliations:** 1 Jiamusi College, Heilongjiang University of Chinese Medicine, Jiamusi 154007, China; 2 State Key Laboratory of Bioactive Substance and Function of Natural Medicines, Institute of Materia Medica, Chinese Academy of Medical Sciences and Peking Union Medical College, Beijing 100050, China

**Keywords:** endophytic fungus, *Aspergillus fumigatus*, *Erythrophloeum fordii Oliv.*

## Abstract

A new diketopiperazine alkaloid named spirotryprostatin K (**1**), and five known alkaloids, spiro[5*H*,10*H*-dipyrrolo[1,2-*a*:1′,2′-d]pyrazine-2(3*H*),2′-[2*H*]-indole]-3′,5,10(1′*H*)trione (**2**), 6-methoxyspirotryprostatin B (**3**), pseurotin A (**4**), *N*-β-acetyltryptamine (**5**), and lumichrome (**6**) were isolated from the endophytic fungus *Aspergillus fumigatus.* The structure and the absolute configuration of spirotryprostatin K were established by extensive spectroscopic analyses, acid hydrolysis and ECD calculations. Pseurotin A exhibited indirect anti-inflammatory activity by suppressing the lipopolysaccharide-induced proinflammatory factors in BV2 microglial cells, with an IC_50_ of 5.20 µM.

## 1. Introduction

Interest in endophytic fungi as sources of novel bioactive compounds is increasing because bioactive natural products from endophytic microbes have shown enormous potential as sources of new medicinal and agricultural products [1,2]. Considering that the relationship between endophytic fungi and host plants may range from latent phytopathogenesis to mutualistic symbiosis [1], endophytic fungi residing in poisonous plants may produce structurally diverse secondary metabolites as a consequence of their biological and biochemical evolution. We therefore initiated a research program focusing exclusively on the discovery of secondary metabolites from endophytic fungi associated with poisonous plants.

**Figure 1:**
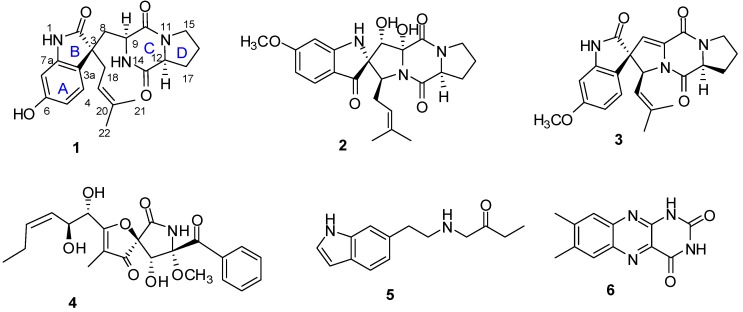
Structures of compounds **1**–‎**6**.

*Erythrophloeum fordii Oliv.* (Leguminosae) is widely distributed in the south of China and its bark and seed have been used in folk medicine to facilitate blood circulation and dispersing blood stasis [3]. We have previously discovered and reported a series of bioactive constituents from this plant [4]. Our efforts have now been directed at investigating the alkaloids found among the secondary metabolites of the endophytic fungus *Aspergillus* *fumigatus* isolated from the plant stem*.* In our study, a new diketopiperazine alkaloid, spirotryprostatin K (**1**), was isolated from the EtOAc extract of a culture of *A.* *fumigatus*, together with the five known alkaloids: spiro[5*H*,10*H*-dipyrrolo-[1,2-*a*:1′,2′-d]pyrazine-2(3*H*),2′-[2*H*]-indole]-3′,5,10(1′*H*)trione (**2**) [5], 6-methoxyspirotryprostatin B (**3**) [6], pseurotin A (**4**) [7], *N*-β-acetyltryptamine (**5**) [8], and lumichrome (**6**) [9]. In this paper, we describe the isolation, structural elucidation, and biological activity of the isolated compounds (Figure 1).

## 2. Results and Discussion

**Figure 2:**
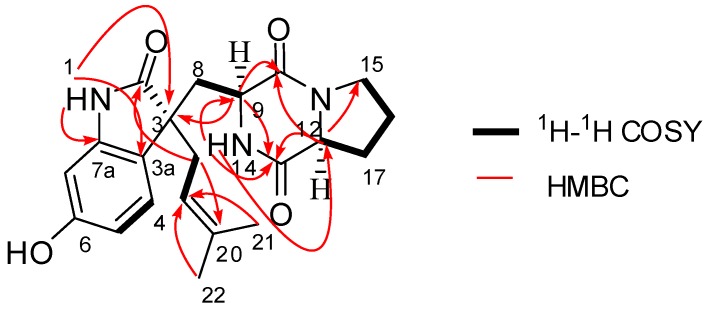
Key ^1^H-^1^H COSY and HMBC correlations of **1**.

Compound **1** was obtained as a yellow amorphous powder and was assigned a molecular formula of C_21_H_25_N_3_O_4_ based on HRESIMS (*m*/*z* 406.1751 [M + Na]^+^; calcd 406.1737) and NMR data (Table 1). The UV spectrum showed maxima ascribable to a substituted benzene ring (213 and 270 nm), and the IR spectrum showed the presence of amide carbonyl (1692 and 1636 cm^−1^) and hydroxyl (3212 cm^−1^) groups. The ^1^H-NMR data (Table 1) revealed a 1,2,4-trisubstituted benzene ring [δ_H_ 7.41 (d, *J* = 8.1 Hz, H-4), 6.94 (dd, *J* = 8.1 and 2.2 Hz, H-5), and 6.91 (d, *J* = 2.2 Hz, H-7)], an olefinic proton, δ_H_ 5.27 (dd, *J* = 7.1 and 7.1 Hz, H-19), and two methyl signals, δ_H_ 1.51 (3H, s, Me-21) and 1.56 (3H, s, Me-22), along with signals due to several methine and methylene groups. The ^13^C-NMR data (Table 1) revealed twenty-one carbon resonances, including three amide carbonyls at δ_C_ 184.0 (C-2), 170.6 (C-10), and 166.1 (C-13), one oxygen-bearing sp^2^ carbon at δ_C_ 159.8. According to the above features, the NMR spectroscopic data of **1** were very similar to those of spirotryprostatin A [10], the major difference between **1** and spirotryprostatin A being that the methine carbon (δ_C_ 60.2, C-18) in spirotryprostatin A was replaced by a methylene carbon (δ_C_ 38.2, C-18) in **1**. The chemical shift changes and analyses of the degrees of unsaturation of **1** and spirotryprostatin A showed that the bond between C-18 and N-14 in spirotryprostatin A was broken in **1**, which was confirmed by the key HMBC correlations from H_2_-18 to C-2/C-20 and from H-14 to C-9/C-12/C-13 (Figure 2). The other difference between **1** and spirotryprostatin A was that the methoxy (δ_C_ 55.5, δ_H_ 3.80) in spirotryprostatin A was replaced by a hydroxyl in **1**.

**Table 1:** ^1^H-NMR and ^13^C-NMR data of **1** (800 MHz for ^1^H, 200 MHz for ^13^C in pyridine-*d*_5_).

Position	δ_C_	δ_H_ (*J* in Hz)	HMBC
1 (NH)		11.96 s	C-2, C-3, C-3a, C-7a
2	184.0		
3	52.8		
3a	122.3		
4	125.6	7.41 d (8.1)	C-3, C-5, C-6, C-3a, C-7a
5	109.8	6.94 dd (8.1, 2.2)	C-4, C-6, C-7, C-3a
6	159.8		
7	99.2	6.91 d (2.2)	C-5, C-6, C-3a, C-7a
7a	144.7		
8	36.4	3.62 dd (15.0, 1.9) 2.50 dd (15.0, 8.4)	C-2, C-3, C-18, C-9, C-10
9	53.3	4.06 dd (8.4, 1.9)	C-3, C-8, C-10, C-13
10	170.6		
12	59.2	3.87 t (8.2)	C-10, C-13, C-15, C-16, C-17
13	166.1		
14 (NH)		7.81 s	C-9, C-10, C-12, C-13
15	45.9	3.50 dt (11.3, 8.1) 3.37 m	C-10, C-12, C-17
16	23.2	1.63 m 1.53 m	C-12, C-15, C-17
17	28.4	2.10 m	C-12, C-14, C-15, C-16
18	38.2	2.84 dd (14.1, 7.1) 2.78 dd (14.1, 7.1)	C-2, C-3, C-4, C-8, C-20
19	118.9	5.27 dd (7.1, 7.1)	C-3, C-18, C-20, C-21, C-22
20	135.9		
21	26.1	1.51 s	C-19, C-20, C-22
22	18.3	1.56 s	C-19, C-21, C-22

**Figure 3:**
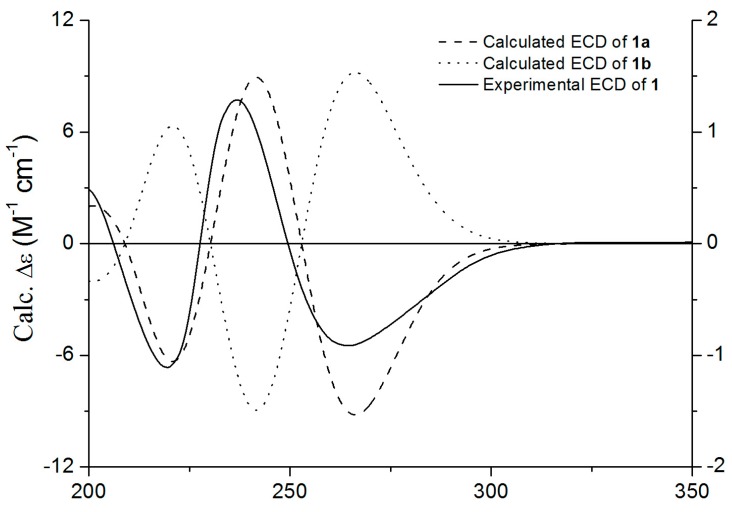
Experimental and calculated ECD spectra of **1**.

The relative configurations of C-9 and C-12 were deduced from the NOESY and NOE spectra (Figures S8 and S9 in Supporting Materials). NOESY correlation of H-9 with H-12 indicated that H-9 and H-12 were on the same face of ring C. A strong NOE was observed for H-12 after irradiation of H-9, which also indicated that H-9 and H-12 were on the same face of ring C. Marfey’s method [10] was applied to assign the absolute configuration of the proline residue resulting from acid hydrolysis of **1**. HPLC analysis of the 1-fluoro-2,4-dinitrophenyl-5-L-alanine amide (FDAA) derivatives of the acid hydrolysate of **1** gave the same retention time as that prepared from sample of authentic L-proline (Figures S13–‎S15 in Supplementary Materials). Therefore, the proline residue in **1** was assigned the L-configuration. Considering the relative configurations established between C-9 and C-12 by NOESY and NOE data, the absolute configuration of C-9 was determined to be *S*. According to the above analyses, the possible absolute configurations of **1** were proposed to be (3*S*, 9*S*, 12*S*) (**1a**) or (3*R*, 9*S*, 12*S*) (**1b**). The calculated ECD spectrum of **1a** displayed a CD curve similar to the experimental spectrum of **1** (Figure 3). Thus, the structure of **1** was determined as shown in Figure 1, and named spirotryprostatin K.

The known compounds, spiro[5*H*,10*H*-dipyrrolo-[1,2-*a*:1′,2′-*d*]pyrazine-2(3*H*),2′-[2*H*]-indole]- 3′,5,10(1′*H*)trione (**2**), 6-methoxyspirotryprostatin B (**3**), pseurotin A (**4**), *N*-β-acetyltryptamine (**5**), and lumichrome (**6**) were isolated and identified by comparing their experimental physical and spectroscopic data with literature values [5,6,7,8,9].

The isolated compounds **4** indirectly exhibited anti-inflammatory activity by suppressing lipopolysaccharide-induced NO production in mouse macrophages with IC_50_ values of 5.20 μM, Dexamethasone was used as the positive control, with IC_50_ = 2.5 × 10^−2^ μM. The other isolated compounds **2**–‎**6** were inactive (IC_50_ > 10 μM) for the inhibition of NO production. 

## 3. Experimental Section

### 3.1. General Information

UV spectra were measured on a JASCO V650 spectrophotometer (Jasco, Tokyo, Japan). CD spectra were recorded on a JASCO J-815 spectropolarimeter (Jasco, Easton, MD, USA). IR spectra were recorded on a 5700 FT-IR spectrometer (Nicolet, Madison, WI, USA). HRESIMS data was recorded on a 6250 Accurate-Mass Q-TOF LC/MS spectrometer (Agilent, Santa Clara, CA, USA). NMR spectra were recorded on a Bruker 800 spectrometer (Bruker, Ettlingcn, Germany), for 1D and 2D NMR. Optical rotation was measured on a Jasco P-2000 automatic digital polarimeter (Jasco, Tokyo, Japan). Preparative HPLC was performed on an LC-6AD instrument (Shimadzu, Kyoto, Japan) equipped with an SPD-10A detector (Shimadzu, Kyoto, Japan) using an YMC-Pack ODS-A column (250 × 20 mm, 5 µm). Column chromatography (CC) was performed on silica gel (200–300 mesh, Qingdao Marine Chemical Inc., Qingdao, China).

### 3.2. Fungal Material

The fungus *Aspergillus fumigatus* was separated from the stem of *Erythrophloeum fordii Oliv*. by Jun-Gui Dai of the Institute of Materia Medica, Chinese Academy of Medical Sciences & Peking Union Medical College, and identified by Xian-Zhi Jiang of the Institute of Microbiology, Chinese Academy of Sciences (Beijing, China). 

### 3.3. Extraction and Isolation

The liquid fermentation was applied to a D101 macroporous resin (eluted with H_2_O and 95% EtOH), and then the 95% EtOH fraction (1050 g) was extracted with EtOAc and *n*-butanol. The EtOAc extract (100 g) was subjected to silica gel column chromatography, eluting with CH_3_OH/CH_2_Cl_2_, to yield six fractions (FrA-F). Fraction E (7.6 g) was subjected to Sephadex LH-20 column chromatography eluting with MeOH to give ten fractions FrE1–‎E10. Fraction E2 (1.3 g) was separated by MPLC with MeOH/H_2_O (10%−95% MeOH in H_2_O), then further purified by preparative HPLC with CH_3_CN/H_2_O (20:80) to obtain compound **1** (10 mg), **2** (12 mg), **3** (10 mg), and with CH_3_CN/H_2_O (30:70) to obtain **4** (9 mg) and **5** (6 mg). Fraction E6 (1.6 g) subjected to Sephadex LH-20 colunm chromatography eluting with MeOH to give **6** (10 mg).

### 3.4. Product Characterization

*Spirotryprostatin K* (**1**): white, amorphous power; ${\left[\alpha \right]}_{D}^{25}$ −50.1 (*c* 0.1, CH_3_OH); IR ν_max_ 3212, 2922, 1692, 1636, 1434, 1203, 1028 cm^−1^; UV (MeOH) λ_max_ (log ε): 202 (4.19), 213 (4.09), 270 (2.10); CD (MeOH) nm (Δε): 219 (−1.1), 236 (+1.28), 264 (−0.91); ^1^H-NMR and ^13^C-NMR see Table 1; HRESIMS *m/z* 406.1751 [M + Na]^+^ (calcd for 406.1737, C_21_H_25_ N_3_O_4_Na).

### 3.5. Absolute Configuration of 1 

An amino acid (l-proline or d-proline standard, 0.2 mg, Sigma, St. Louis, MO, USA) was dissolved in H_2_O (30 μL) and treated with 1% 1-fluoro-2,4-dinitrophenyl-5-l-alanine amide (FDAA) in acetone (60 μL) and 6% Et_3_N in 30 μL of H_2_O at 40 °C for 1 h. After cooling to room temperature, the derivative was analyzed by reversed-phase HPLC with detection by UV absorption at 340 nm. The column [Agilent C18, 5 μm, 4.6 × 150 mm] was eluted with 35% CH_3_CN in H_2_O (0.1% trifluoroacetic acid) for 30 min. The standards gave the following retention times: 20.17 min for l-proline, and 23.90 min for d-proline. Compound **1** (1 mg) was hydrolyzed in 6 HCl (0.5 mL) and heated at 150 °C in a sealed vial for 1.5 h to yield the corresponding amino acids. The cooled reaction mixture was evaporated to dryness under reduced pressure, and HCl was removed from the residual acid hydrolysate by repeated evaporation from frozen H_2_O (1 mL). The amino acid mixture was then treated in the same manner as the standards above (1% FDAA and 6% Et_3_N). The mixture of FDAA derivatives was filtered, and the filtrate was diluted with H_2_O and analyzed by HPLC. The FDAA derivative of the amino acid liberated from **1** showed the peak at 20.14 min, matching the retention time of l-proline [11].

### 3.6. In Vitro Anti-Inflammatory Activity Assays

C57BL6/J mouse macrophages were cultured in 48-well plates in RPMI1640 medium at 37 °C for 24 h. Then, the cells were divided into four groups: the blank control (RPMI1640 medium only), the LPS control (1 μg/mL LPS in RPMI1640 medium), the experimental control (1 μg/mL LPS and the candidate compounds in RPMI1640 medium), and the positive control (10^−6^ M dexamethasone in RPMI1640 medium). The cells were then incubated at 37 °C for an additional 24 h. From each well, a total of 100 μL of the supernatant was mixed with the same amount of Griess reagent, and the absorbance value was measured at 570 nm using a microplate reader. Sodium nitrite was used as the standard to calculate the NO_2_^−^ concentration [12,13].

## 4. Conclusions

A new diketopiperazine alkaloid, spirotryprostatin K (**1**), was isolated from the endophytic fungus *Aspergillus fumigatus.* Its structure and absolute configuration were determined by a combination of extensive spectroscopic methods, acid hydrolysis, and ECD calculations. A known alkaloid, pseurotin A (**4**) exhibited indirect anti-inflammatory activity by suppressing the lipopolysaccharide-induced proinflammatory factors in BV2 microglial cells, with an IC_50_ of 5.20 μM.

## Supplementary Materials

Supplementary materials can be accessed at: http://www.mdpi.com/1420-3049/20/06/10793/s1.
