# Supplementary material for: Metabolites Produced by the Endophytic Fungus Aspergillus fumigatus from the Stem of Erythrophloeum fordii Oliv
Source: Molecules. 2015 Jun 11;20(6):10793–9. doi: 10.3390/molecules200610793 (PMC6272661; doi:10.3390/molecules200610793)
Supplement: Supplementary file 1 [file molecules-20-10793-s001.pdf]

Supplementary Materials

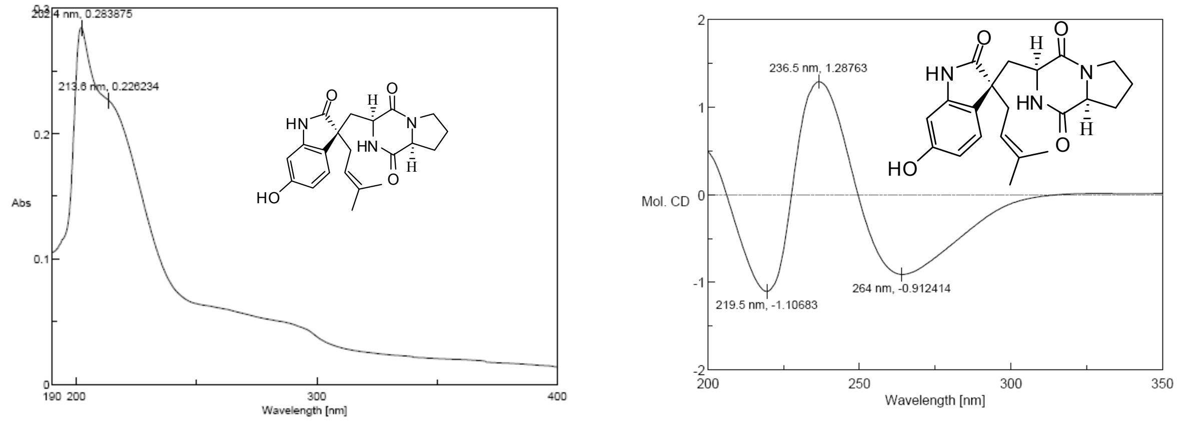

Figure S1. UV and CD spectra of compound **1** in MeOH.

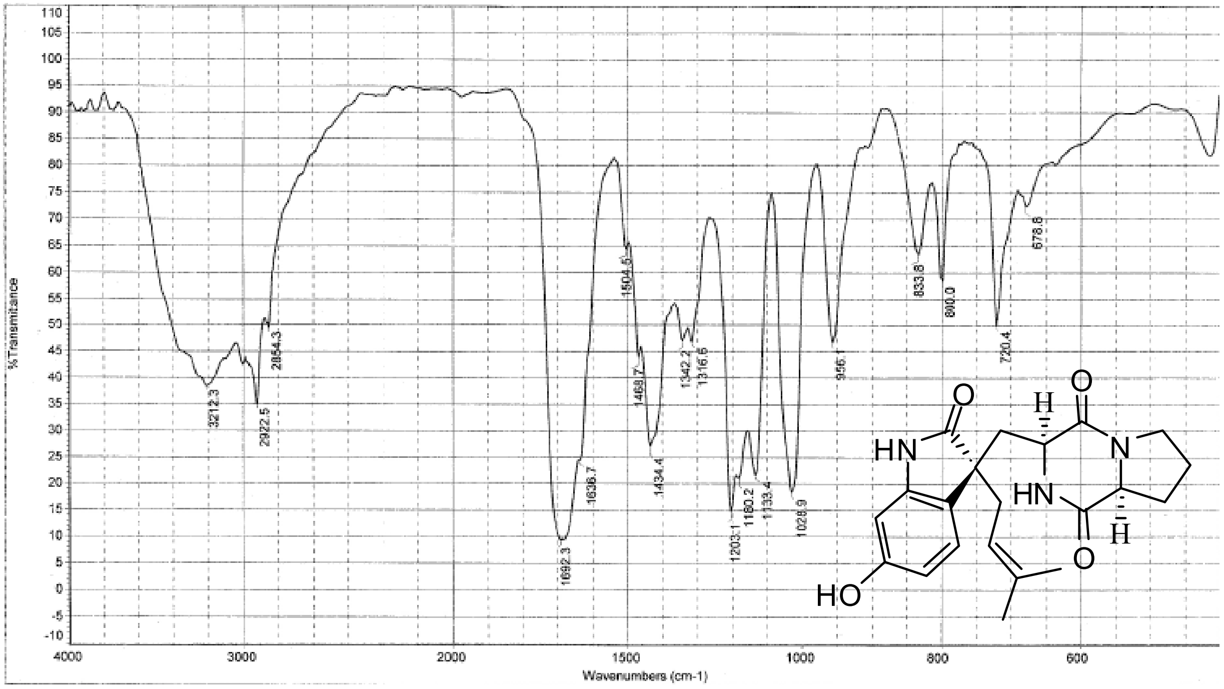

Figure S2. IR spectrum of compound **1**.

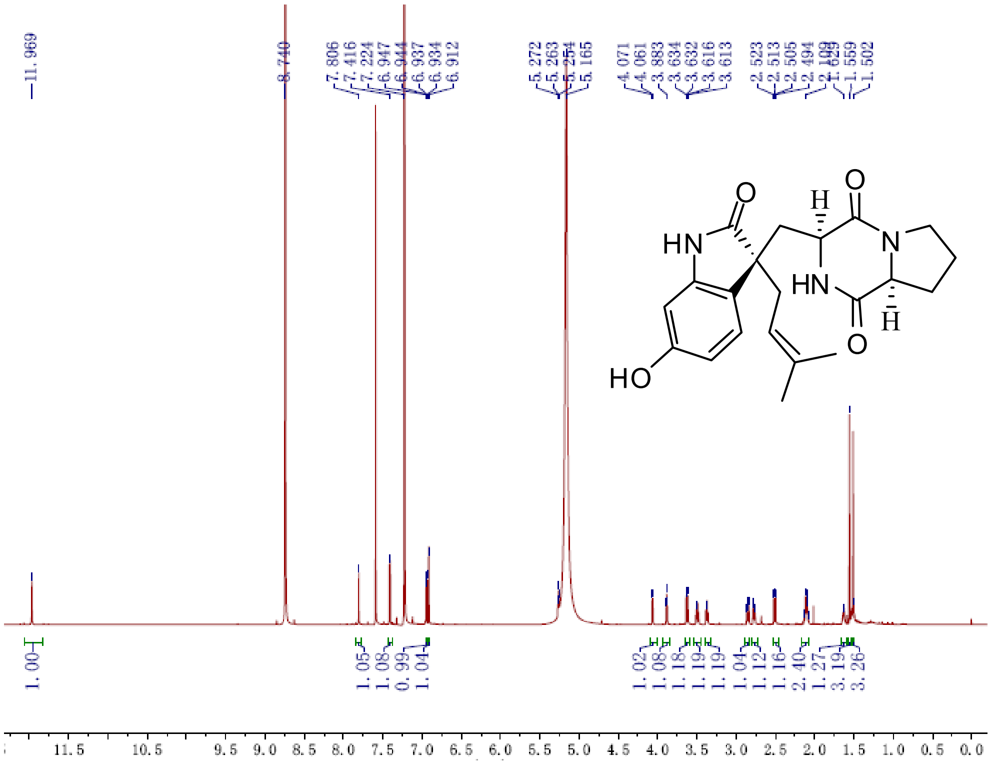

Figure S3. ^{1}H-NMR spectrum of compound **1** in pyridine-*d*5 (800 MHz).

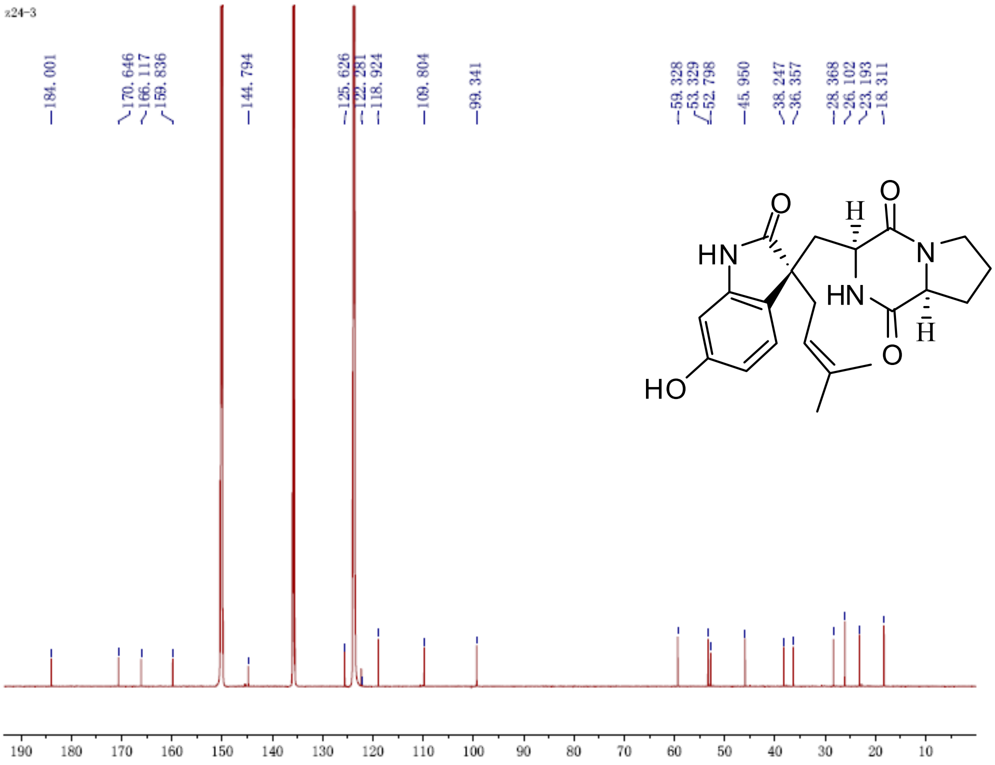

Figure S4. ^{13}C-NMR spectrum of compound **1** in pyridine-*d*5 (200 MHz).

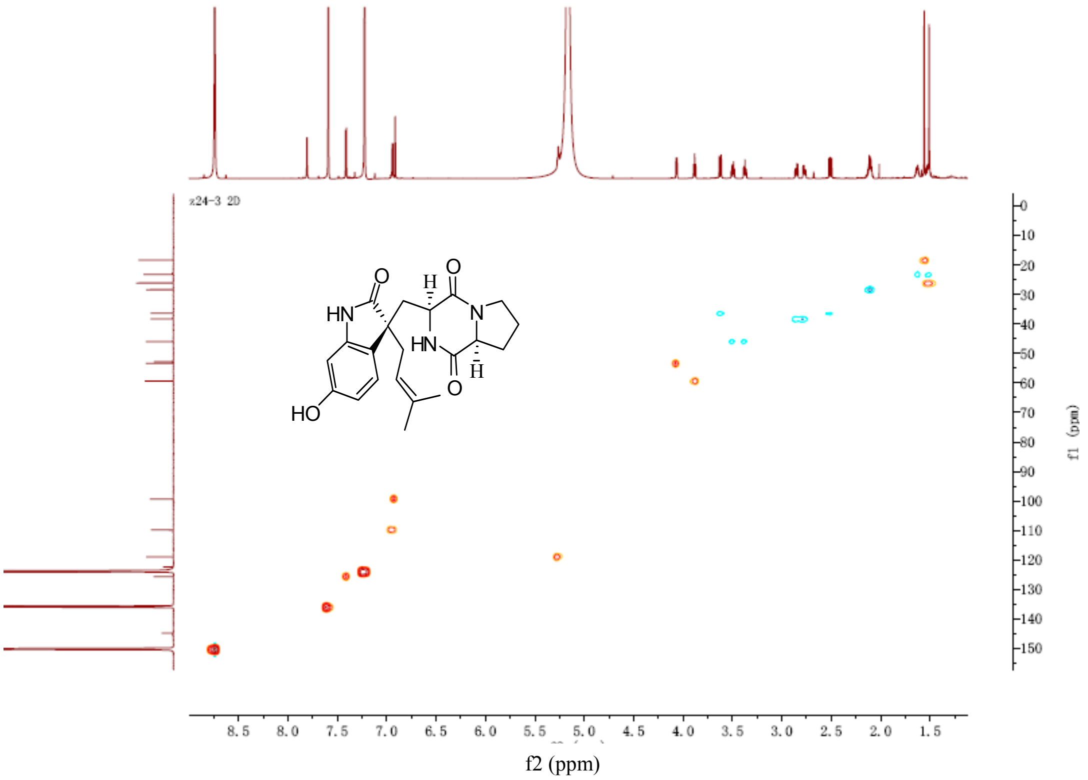

Figure S5. HSQC spectrum of compound **1** in pyridine-d_5 (800 MHz).

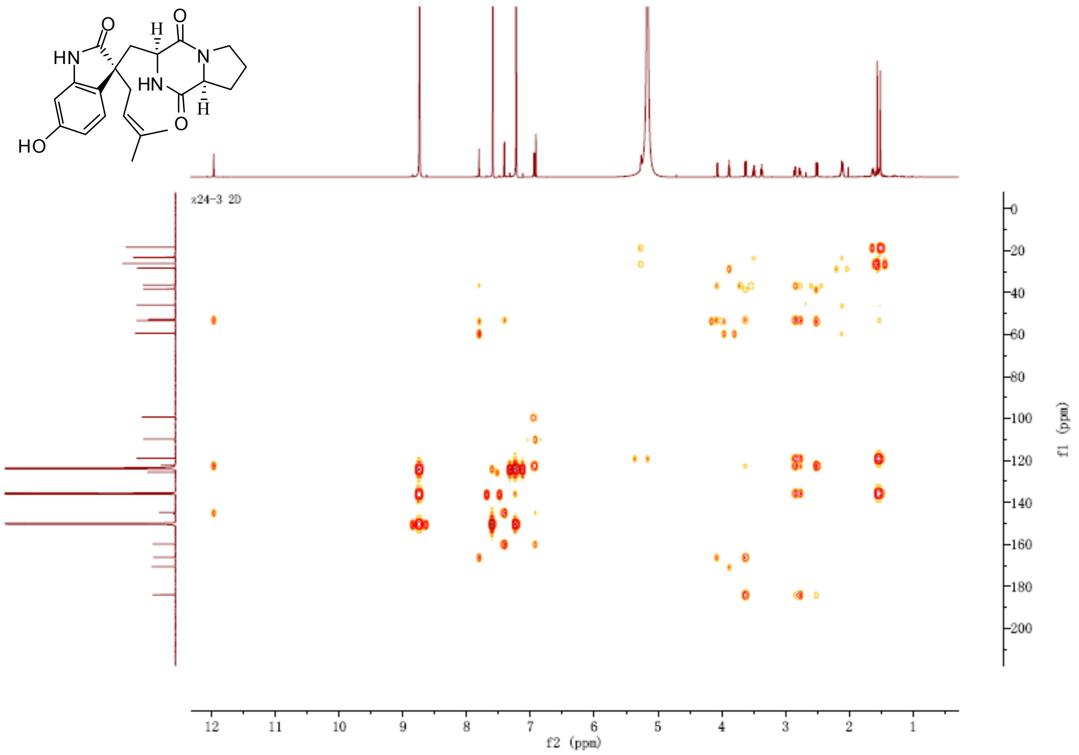

Figure S6. HMBC spectrum of compound **1** in pyridine-d_5 (800 MHz).

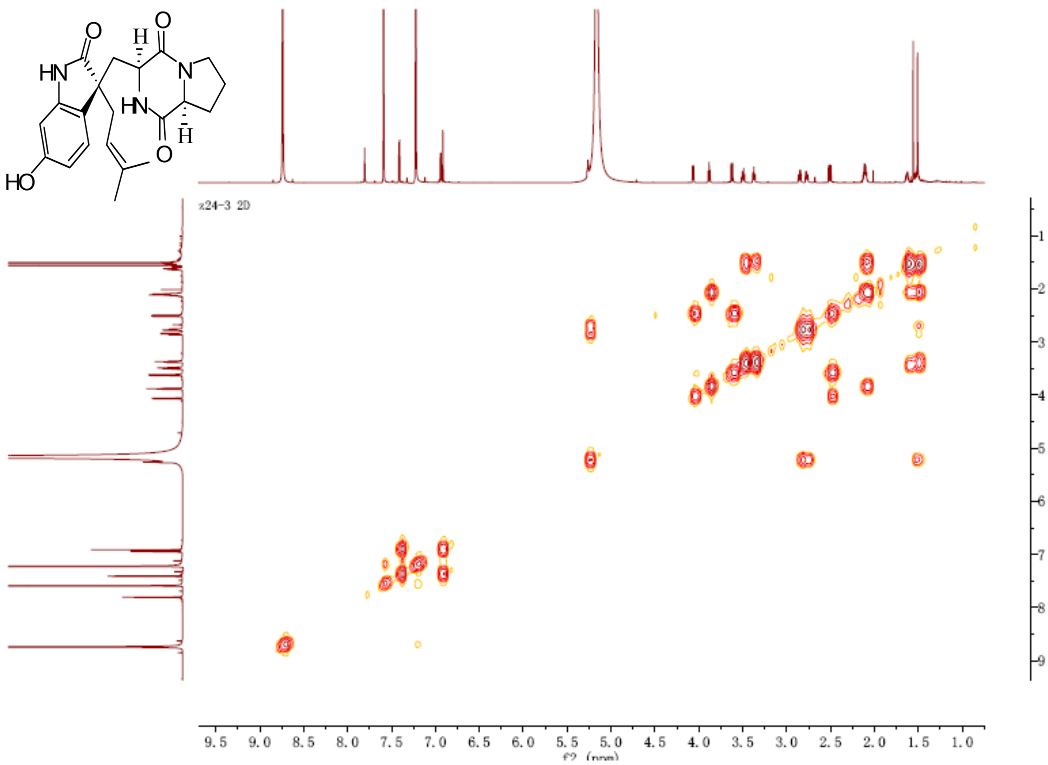

Figure S7. ^{1}H-^{1}H COSY spectrum of compound **1** in pyridine-d_5 (800 MHz).

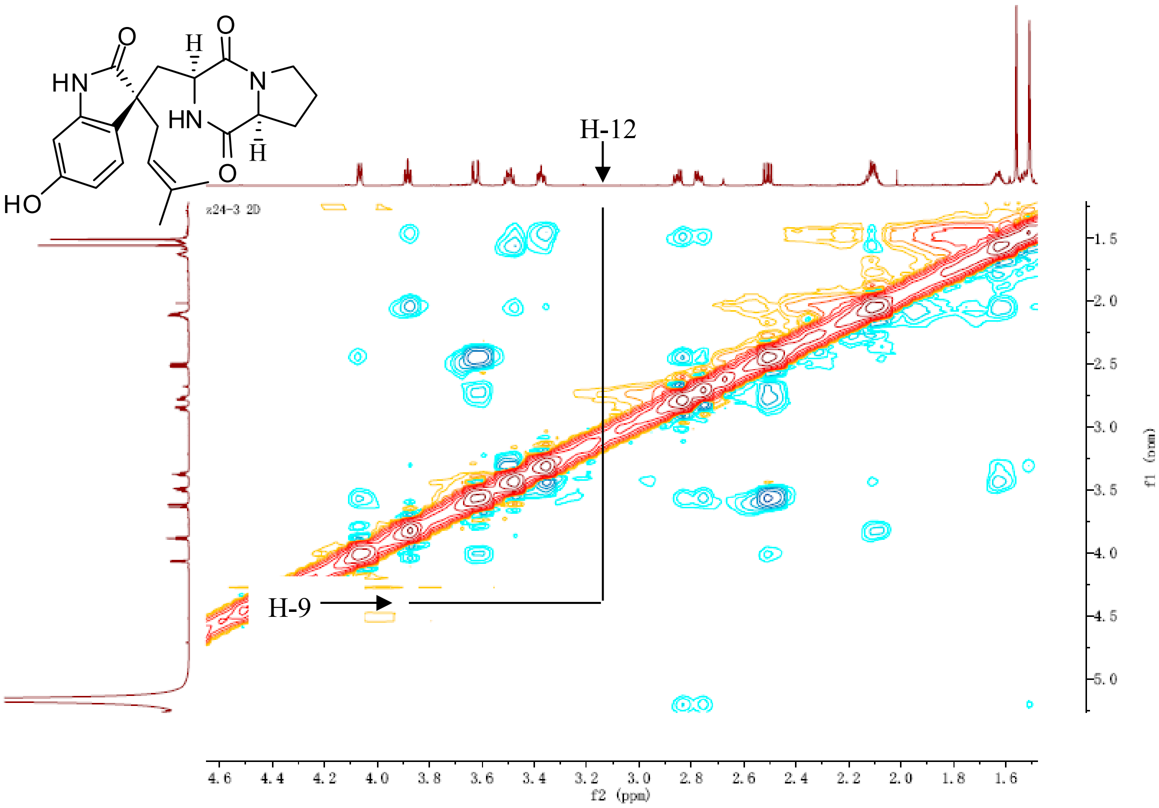

Figure S8. NOESY spectrum of compound **1** in pyridine-d_5 (800 MHz).

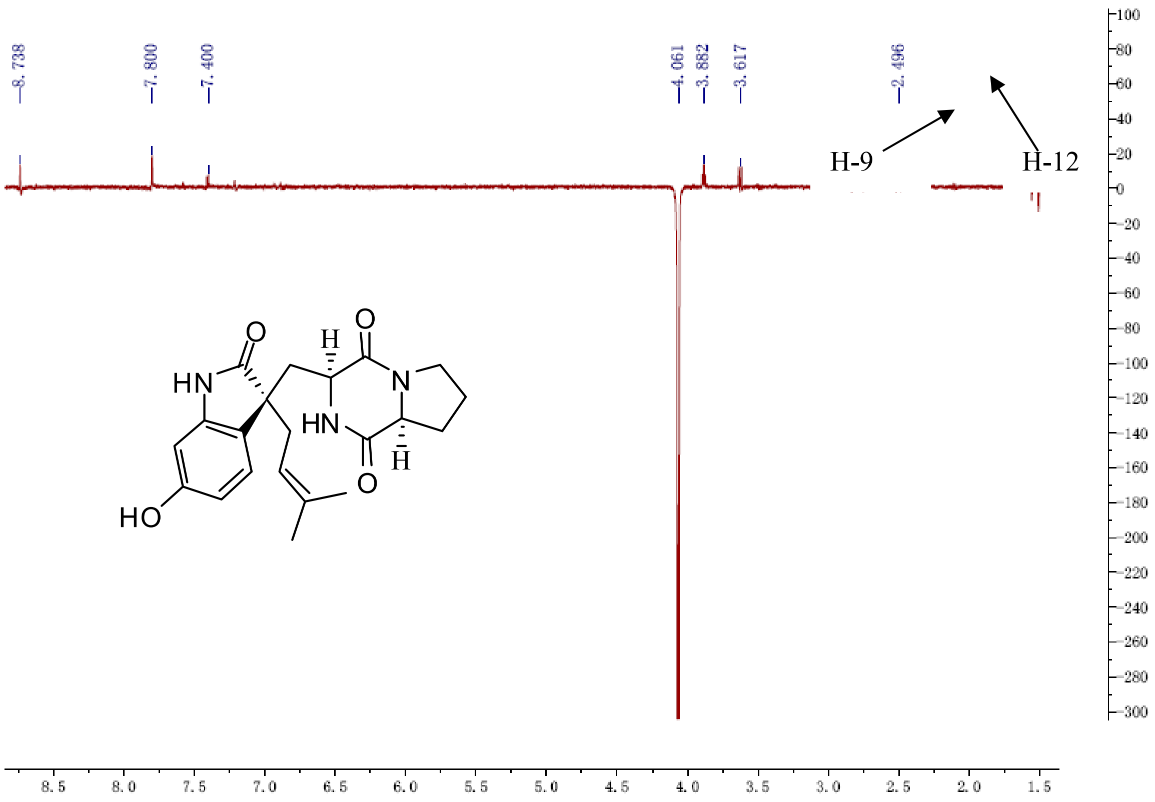

Figure S9. NOE spectrum of compound **1** in pyridine-d_5 (800 MHz).

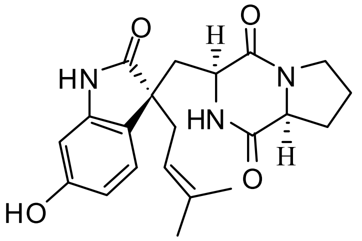

m/z	Ion	Formula	Abundance
406.1751	(M+Na)+	C21 H25 N3 Na O4	750628.6

Best	Formula (M)	Ion Formula	Calc m/z	Score	Cross S	Mass	Calc Mass	Diff (ppm)	Abs Diff (ppm)	Abund Match	Spacing Mat	Mass Match	m/z	DBE
☑	C21 H25 N3 O4	C21 H25 N3 Na O4	406.1737	99.74		383.1858	383.1845	-3.4	3.4	99.94	99.67	99.66	406.1751	11
☐	C26 H25 N O2	C26 H25 N Na O2	406.1778	98.84		383.1858	383.1885	7.11	7.11	98.77	99.55	98.52	406.1751	15
☐	C18 H29 N3 O4 S	C18 H29 N3 Na O4 S	406.1771	98.33		383.1858	383.1879	5.39	5.39	95.94	99.56	99.15	406.1751	6
☐	C13 H29 N5 O6 S	C13 H29 N5 Na O6 S	406.1731	96.8		383.1858	383.1839	-5.12	5.12	90.38	99.63	99.23	406.1751	2

Figure S10. HRESIMS spectrum of compound **1**.

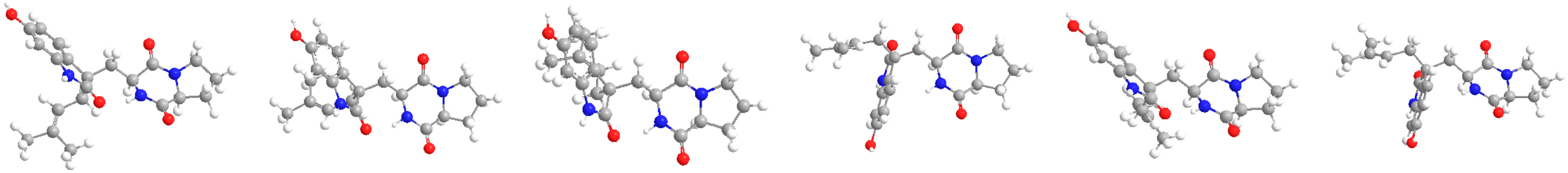

1aC1 (40.289%) 1aC2 (27.313%) 1aC3 (9.136%) 1aC4 (6.454%) 1aC5 (6.326%) 1aC6 (4.441%)

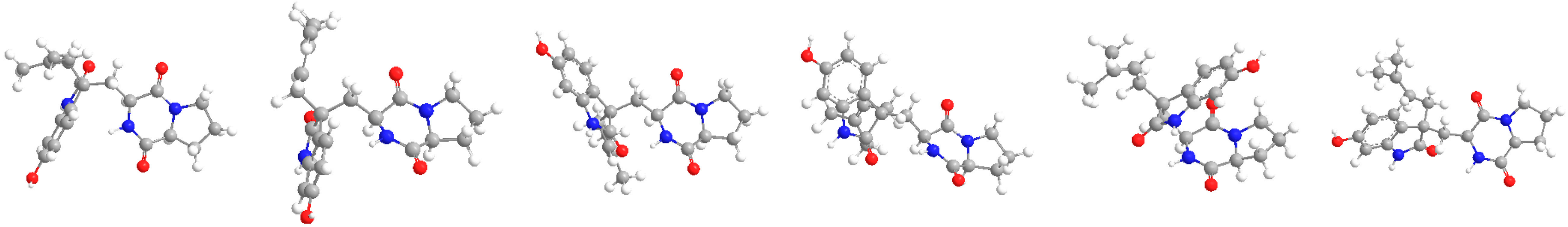

1aC7 (2.022%) 1aC8 (1.743%) 1aC9 (0.973%) 1aC10 (0.427%) 1aC11 (0.228%) 1aC12 (0.159%)

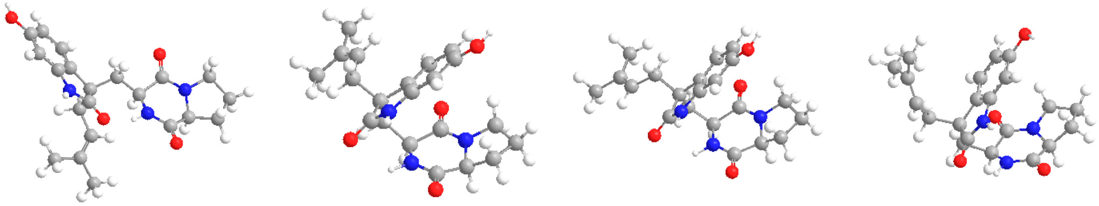

1aC13 (0.149%) 1aC14 (0.143%) 1aC15 (0.136%) 1aC16 (0.061%)

Figure S11. Geometries of **1** at the B3LYP/6–31G (d) level in methanol.

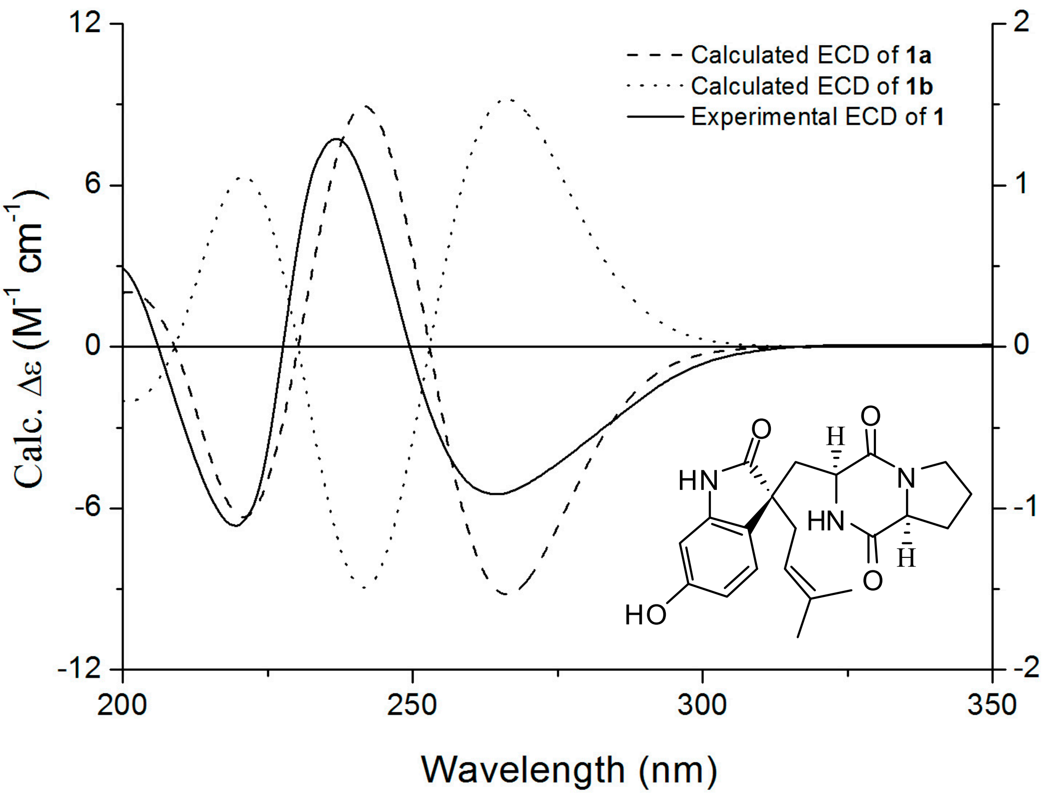

Figure S12. Comparison of the experimental and calculated ECD spectra of **1.**

Computational details. All quantum-chemical calculations have been performed at 298K on (3*S*, 9*S*, 12*S*) configuration of **1** by the Gaussian09 program package [1]. A system conformational analysis was employed using the MMFF94 force field via the MOE software package [2,3] The only resultant conformer was further optimized and checked as the true minima of potential energy surface by the density functional theory method at the 6-31G (d) basis set level. Conductor-like polarizable continuum model (CPCM) was adopted to consider solvent effects using the dielectric constant of methanol ($\varepsilon = 32.6$). The 30 lowest electronic transitions were calculated and rotational strengths of each electronic excitation were given using both dipole length (R_{len}) and dipole velocity (R_{vel}) representations. The rotational strengths in R_{vel} form were converted to a Gaussian-type curve with a half-bandwidth of 0.30 eV. ECD spectrum was compared with the experimental ECD data of **1** (Figure S12).

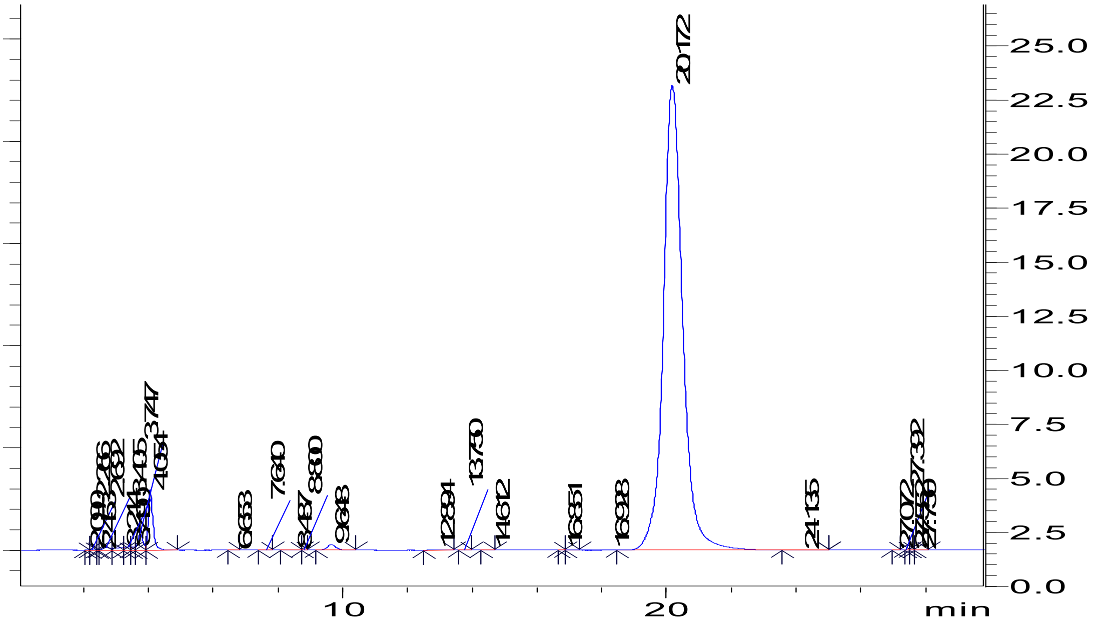

Figure S13. HPLC analysis of the FDAA derivative of standard L-Proline.

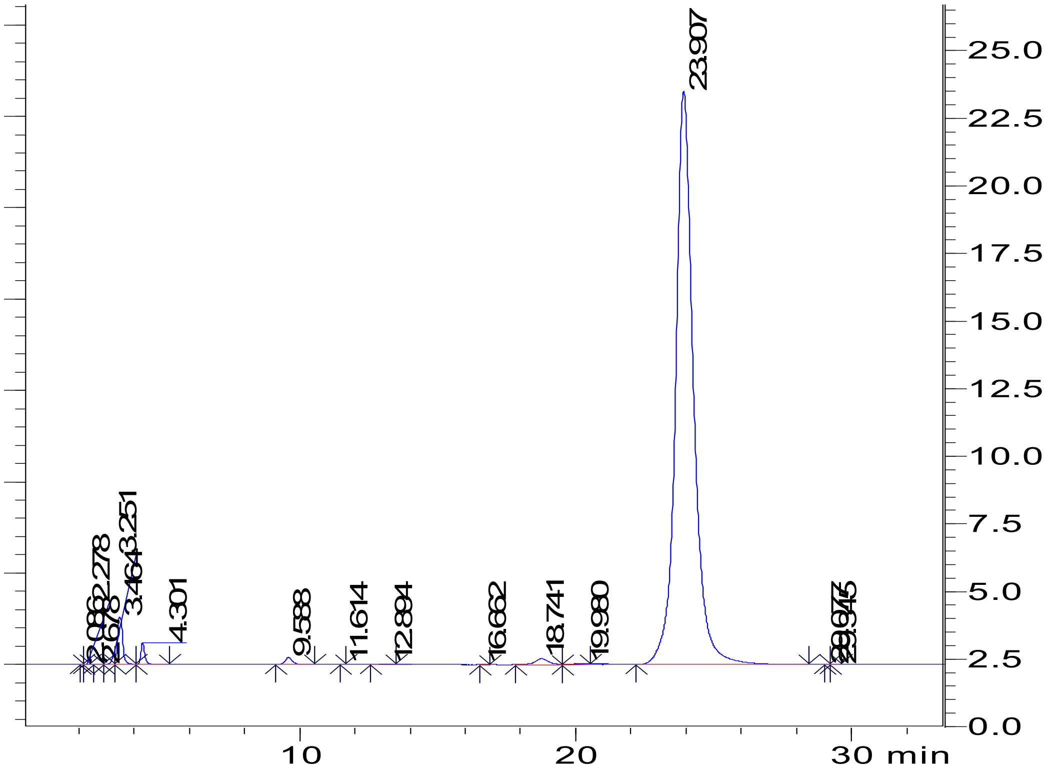

Figure S14. HPLC analysis of the FDAA derivative of standard D-Proline.

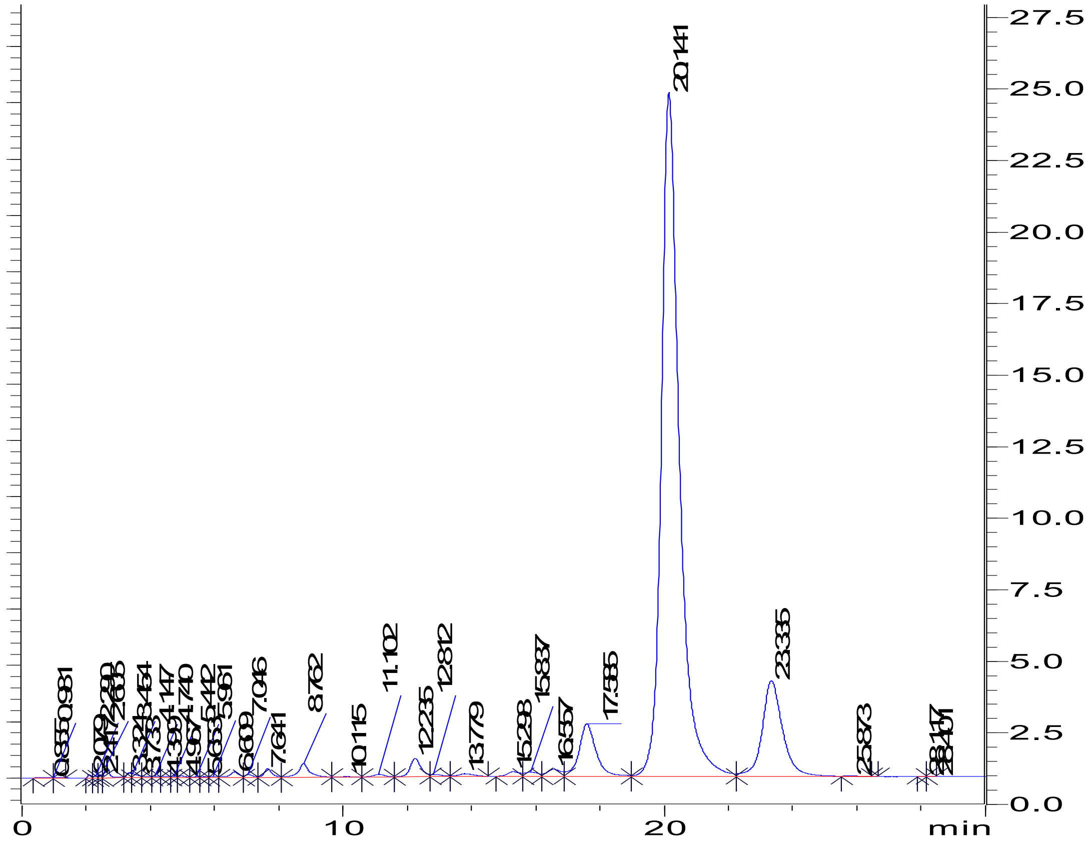

Figure S15. HPLC analysis of the FDAA derivatives of the acid hydrolyzate of **1.**

References

1. Frisch, M.J.; Trucks, G.W.; Schlegel, H.B.; Scuseria, G.E.; Robb, M.A.; Cheeseman, J.R.; Scalmani, G.; Barone, V.; Mennucci, B.; Petersson, G.A.; *et al. Gaussian 09*; Revision D. 01; Gaussian, Inc.: Wallingford, CT, USA, 2009.
2. *MOE2009.10*; Chemical Computing Group Inc.: Montreal, QC, Canada.
3. Berova, N.; Lorenzo, D.B.; Gennaro, P. Application of electronic circular dichroism in configurational and conformational analysis of organic compounds. *Chem. Soc. Rev.* **2007**, *36*, 914–931.
